# Austrian Pompe Outcome Consensus (APOC): a national Delphi study

**DOI:** 10.1186/s13023-025-04187-0

**Published:** 2026-01-16

**Authors:** Florian B. Lagler, Thomas Scherer, Jörg Weber, Martina Huemer, Wolfgang Löscher

**Affiliations:** 1 Institute for Inborn Errors of Metabolism and Department of Pediatrics, Paracelsus Medical University, Salzburg, Austria; 2 Division of Endocrinology and Metabolism, Department of Internal Medicine III, Medical University of Vienna, Währinger Gürtel 18-20, Vienna, 1090 Austria; 3 Department of Neurology, Klinikum Klagenfurt, Feschnigstraße 11, Klagenfurt am Wörthsee, 9020 Austria; 4 Department of Paediatrics, Landeskrankenhaus Bregenz, Bregenz, Austria; 5 Division of Metabolism, Children’s Research Centre, University Children’s Hospital Zurich, Steinwiesstrasse 75, Zürich, 8032 Switzerland; 6 Department of Neurology, Medical University Innsbruck, Anichstrasse 35, Innsbruck, 6020 Austria

**Keywords:** Pompe disease, Delphi consensus, Follow-up assessments, Enzyme replacement therapy, AWMF S2k guidelines, Austria

## Abstract

**Background:**

Follow-up assessments form the basis for the continuous optimization of therapy and supportive care on an individual level, for confirming treatment efficacy, and for detecting newly emerging or unexpectedly progressive symptoms early enough to permit timely therapeutic intervention. For Pompe disease, evidence based guidelines on which assessments should constitute the minimum standard and which are required in specific situations only, were missing. Therefore, we started the Austrian Pompe Outcome Consensus (APOC) Study.

**Methods:**

APOC was a Delphi process with two classical online and a modified third round, implemented September 2023–May 2024, following the AWMF S2k guideline. A five-member interdisciplinary steering committee invited 23 clinical experts, achieving response rates of 69.6% and 100%. A questionnaire was developed via literature scoping and an expert workshop. The importance and recommended frequency of follow-up assessments were rated using AGREE II consensus thresholds, and the classification for recommendation strength of the German Association of the Scientific Medical Societies (AWMF;Arbeitsgemeinschaft der Wissenschaftlichen Medizinischen Fachgesellschaften e. V.).

**Results:**

34 statements achieved consensus. Strong recommendations included the 6-minute walk test (6MWT), timed tests, Pompe PEDI and other age-appropriate functional tests in children, muscle tests, handheld dynamometry, Fatigue Severity Scale, patient-reported outcome measures (e.g. R-Pact), forced vital capacity (sitting/supine), morphologic muscle imaging studies, pain and quality of life assessment. Further recommendations included respiratory (MIP/MEP) and sleep studies (polysomnography), creatine kinase, antibody titers, swallowing studies, liver sonography, hearing tests and speech and speech/oromotor function, physical therapy and rehabilitation, bone density assessment, and caregiver psychosocial care.

**Conclusions:**

The APOC Delphi consensus yields AGREE II–compliant, systematically weighted recommendations delineating essential and optional follow-up assessments for Pompe disease in the context of Austrian healthcare. The applied method enabled a structured and efficient consensus-building process and appears well suited for addressing comparable questions in other rare disease contexts.

**Clinical trial number:**

Not applicable.

## Background

Pompe disease, or glycogenosis type II, is an autosomal-recessive disorder caused by pathogenic variants in the *GAA* gene leading to deficiency of the lysosomal enzyme acid α-glucosidase and consequent intralysosomal glycogen accumulation. Based on the genotype Pompe disease spans a phenotypic spectrum from rapidly fatal infantile-onset disease—with cardiomyopathy and profound muscle weakness—to late-onset presentations characterized by slowly progressive skeletal and respiratory muscle decline. The estimated prevalence is approximately 1: 350,914 in Austria and 1: 283,000 across Europe [1, 2].

Severely affected patients face a significant burden of disease. Respiratory insufficiency and acute respiratory emergencies represent the leading causes of morbidity and mortality, alongside axial and limb-girdle muscle weakness, cardiomyopathy, arrhythmias, feeding and swallowing dysfunction, and pervasive fatigue. Such multisystem involvement mandates an interdisciplinary, personalized management approach to optimize clinical outcomes [3–5].

Prospective, predominantly annual follow-up assessments have become the standard of care. Encompassing four core domains—functional tests, patient-reported outcome measures, imaging, and biomarkers—they enable early detection of disease progression and timely intervention. However, despite recognition of the importance of these various assessments, there is no consensus on which measures constitute the minimum essential clinical routine versus those of primarily academic interest [4, 5].

This need has been accentuated by therapeutic advances: alglucosidase alfa has served as the cornerstone of enzyme replacement therapy for nearly 20 years, and more recently avalglucosidase alfa and cipaglucosidase-alfa/miglustat have gained approval. Gene therapy approaches are on the horizon. These developments heighten the imperative for a comprehensive yet feasible set of follow-up tests [3].

To address this gap from a national vantage, we embarked on a Delphi-consensus project (DP) in Austria. Our aim was to standardize routine follow-up assessments to facilitate comparability of patient outcomes and provide an objective reference framework for discussions with payers and health authorities. We chose the AWMF S2k guideline methodology for its structured, transparent consensus thresholds and efficiency. In this DP with two classic online and a modified third round expert input was systematically harnessed to define minimum and optional montitoring standards [6, 7].

## Methods

This report is written in accordance with the DELPHISTAR publication standard [8].

### Study design and framework

This national consensus project adopted a classic Delphi design embedded within the AWMF S2k guideline framework [7] to develop minimum and optional follow-up assessments for Pompe disease monitoring in Austria. The study was conducted from September 2023 to May 2024.

### Funding and support

The study was financially and logistically supported by Sanofi-Aventis GmbH (Vienna, Austria) Sanofi had no influence on study design, data collection, analysis, interpretation, or reporting.

### Steering committee

An interdisciplinary team of 5 Pompe specialists with expertise in pediatrics and pediatric metabolic medicine, pharmacology, psychology, neurology and neuromuscular medicine from five Austrian medical centers constituted the steering committee. Scientific process support and documentation were provided by the contract research organization Salzburg Research.

### Expert panel composition

A further 18 experts with experience in managing Pompe patients, were invited. As only a few clinicians and centers in Austria provide care for patients with Pompe disease, no comprehensive inclusion or exclusion criteria were defined for the selection of experts. Instead, all clinicians who are involved in patient care and who have actively participated in educational activities and scientific discussions in the country in the past were invited to take part. Experts covered neurology, pediatrics, metabolic medicine, respiratory care, nursing, physiotherapy and represented all centers. Sixteen experts responded in Round 1 (69.6% response rate) and were re-invited for Round 2 (100% response rate). Steering committee and expert panel members were provided with the current literature on follow-up assessments (see supplement 1).

### Handling of non-integrated perspectives

We proactively invited additional disciplines (e.g., patient representatives, allied health professionals) to participate. Several invitees declined, citing lack of preparedness; therefore, lay patient representatives were excluded from Delphi rounds but subsequently invited to review and endorse the final consensus statements.

### Questionnaire development

The survey instrument was developed by the steering committee following a systematic scoping of current literature and in consultation with Delphi experts at a preparatory workshop (8 November 2023). Clinically relevant domains were identified and prioritized separately for pediatric and adult populations. Items were formulated and graduated in accordance with the AGREE II [9] instrument to ensure clarity and consistency.

### Delphi rounds

Three Delphi rounds were conducted:

- **Online Round 1:** Experts provided initial ratings and qualitative feedback.
- **Online Round 2:** Anonymized, aggregated feedback (means, standard deviations, frequency distributions, and summary comments) was provided; experts re-rated each item, aiming for ≥ 75% agreement per item.
- **Modified Round 3:** The steering committee discussed the results from the anonymous online survey rounds 1 and 2, and an attempt was made to derive a representative statements for each item. Since on certain items only individual experts provided feedback even after two rounds of the online questionnaire, we assumed that a third online round would not yield any additional information. We therefore evaluated these items in a modified round with the experts from the Steering Committee, as they were able to provide qualified assessments on all items. Although the expert group in this step was smaller, we considered it valid because all relevant disciplines and centers were represented in the Steering Committee, and the larger expert panel had already been consulted on these topics in rounds 1 and 2.

Consensus strength followed AGREE II [9] thresholds:

- **Strong consensus:** > 95% agreement
- **Consensus:** 75–94% agreement
- **Majority agreement:** 50–74% agreement
- **No majority agreement:** < 50% agreement

Recommendations were labeled “Should” (strong recommendation), “Should be considered” (recommendation), or “May” (option) in line with AWMF S2k terminology.

### Stopping criteria and modifications

A third classical online round was not conducted because, although the feedback from the preceding rounds was concordant in key aspects, several questions remained unresolved. Therefore, a modified third round was undertaken in which the members of the Steering Committee, representing all participating centers and based on the results of the first two rounds, voted on the final consensus recommendations.

### Data analysis and consensus conference

Quantitative data were analyzed descriptively. Free-text comments underwent thematic analysis to identify and synthesize key insights. Feedback was aggregated across the full expert panel, with subgroup analyses for the pediatric cohort where relevant. Within the 3^rd^ round individual statements (e.g., regarding the frequency of assessments and strength of recommendations) were consolidated into final recommendations. Quantitative measures of consensus stability (such as Kendall’s W or IQR narrowing) were not performed, as they were considered of limited interpretability given the small number of participants.

### Management of bias and dominance effects

The experts completed the questionnaires anonymously and without any influence from the steering committee. The analysis was carried out by an independent agency (Salzburg Research), which also moderated the consensus conferences. This approach minimized the risk of undue influence by individual committee members.

### Illustration of Delphi process

**Fig. 1:**
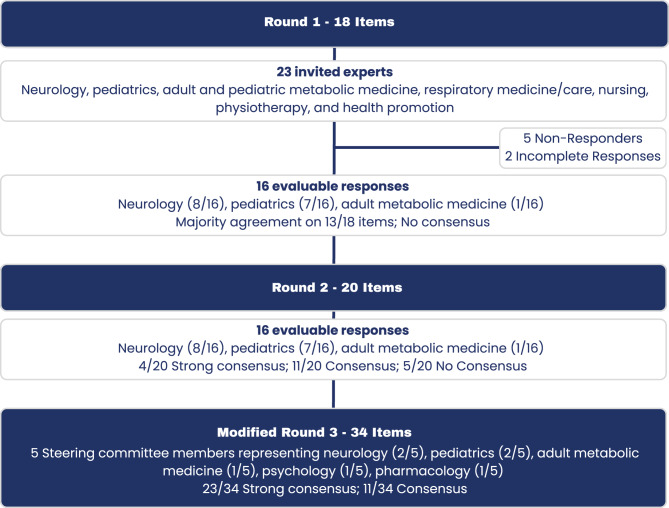
Flow chart of the Austrian Pompe outcome consensus (APOC) Delphi study. The figure depicts the structured three-round Delphi process of the Austrian Pompe outcome consensus (APOC). Round 1 (18 items): of 23 invited clinical experts, 16 (69.6%) provided evaluable responses. Participants represented Neurology, pediatrics, metabolic medicine, respiratory care, nursing, and physiotherapy. Majority agreement was achieved for 13 of 18 statements. Round 2 (20 items): all 16 round-1 participants responded (100%). Based on the 13 items agreed in round-1, 20 statements were derived. Consensus was classified according to agree ii criteria: 4 items reached strong consensus, 11 consensus, and 5 No consensus. Modified round 3 (34 statements): final recommendations were consolidated by a 5-member interdisciplinary steering committee, as a further online round was unlikely to add information. Members represented five Austrian centers and expertise in Neurology (2), pediatrics (2), adult metabolic medicine, psychology, and pharmacology. The 16 statements with (strong) consensus were carried over, and additional 18 statements were phrased based on the feedback on round 2. The outcome comprised 34 consensus statements, with recommendation strength categorized using AWMF S2k terminology (“should,” “should be considered,” “May”) summarized in Table 1

A flow diagram (Fig. 1) details expert recruitment, round participation, and consensus outcomes.

## Results

Figure 1 summarizes the 3 rounds of our Delphi process and Table 1 visualizes the recommendations and agreements gathered within the consensus conference.

**Table 1:** Minimum essential and optional follow-up assessments recommended by the Austrian Pompe outcome consensus (APOC) Delphi study

Neuromuscular Evaluation								Pneumology			Cardiology			Laboratory Markers		
Strongly Recommended (SR)							Optional (O)	SR	Recommended (R)		R		O	R		O
6MWT^1^	Timed Tests	Pompe PEDI^2^	MMT/MRC	Handheld Dynamometry	FSS	PROMs (R-Pact)	Needle EMG	FVC sitting/supine	MIP/MEP	Sleep Studies^3^	ECG, 24 h ECG, Echo	Chest X-Ray	Cardiac MRI	CK	ADA	Hex4
1–2/a	1/a	1/a	regularly and an	regularly and an	regularly and an	1/a	an	1–2/a	1–2/a	1/a	yearly^4^ and an	an	an	regularly and an	an^5^	regularly and an

Radiology				GI & Nutrition					COG-Nition	PAIN	Hearing Speech	QOL	Physio Rehab		Bone	Care Givers
SR	O			R					O	SR	R	SR	R		R	R
Morphologic Muscle Studies	Lung/Diaphragm MRI	Muscle MRI	Skelettal X-Ray	Swallow Studies	Liver US	BW	Nutrition Status	Special Diet	Cognitive/Psycholog Testing	Pain Assessment	Hearing Language Speech Oromotor F.	EQ-5D- 5L HRQoL	Physio	Rehab	Bone Density	Psycho Social Care
an	an	1/a or an	an	an	an	regularly and an	an	an	an	an	an	regularly and an	offer to all	offer if progression	an	offer to all

Recommendations are categorized according to AGREE II consensus thresholds and AWMF S2k terminology: Strength of Recommendation (SR)—strong recommendation (should), recommendation (should be considered), option (may)

Abbreviations: 6MWT, 6-minute walk test; MMT/MRC, Manual Muscle Testing/Medical Research Council Scale; FSS, Fatigue Severity Scale; PROMs, patient-reported outcome measures (e.g. R-Pact); EMG, needle electromyography; FVC, forced vital capacity (sitting/supine); MIP/MEP, maximal inspiratory/expiratory pressures; ECG, electrocardiography; Echo, echocardiography; MRI, magnetic resonance imaging (cardiac, lung/diaphragm, muscle); US, ultrasonography; BW, body weight; CK, creatine kinase; ADA, anti-drug antibodies; Hex4, urinary glucose tetrasaccharide; QOL, quality of life (e.g. EQ-5D-5 L); F., function; an, as needed; 1/a, once per year; 1–2/a, one to two times per year

Notes: ^1^For the 6MWT, testing conditions, cardiopulmonary comorbidities, and timing relative to enzyme replacement therapy should be considered; in pediatric patients, testing should be performed as early as feasible or when cooperative. ^2^Pompe PEDI applies to children only. ^3^Sleep studies include polysomnography with pulse oximetry, transcutaneous capnometry, and morning arterial blood-gas analysis. ^4^Annual frequency applies to ECG only. ^5^Immunological monitoring (ADA) should be considered in case of infusion reactions or clinical deterioration

### Musculoskeletal and neuromuscular evaluation

The 6-minute walk test (6MWT), timed tests, the Pompe-specific Paediatric Evaluation of Disability Inventory (Pompe PEDI), assessments of muscle strength and fatigue, patient-reported outcome measures (PROMs), and age-appropriate assessments for infants and children were strongly recommended. Consensus on 6MWT was reached in round 2, the remaining items have been consented in round 3. Needle electromyography (EMG) was considered optional.

6MWT should be performed 1–2 times per year, taking the following factors into account: a suitable, disturbance-free testing environment, presence of additional cardiopulmonary comorbidities, and time reference to the ERT (e.g., ERT received the previous day). In pediatric patients, the 6MWT should be performed as early as feasible, which can be expected latest by 12 years of age. Further, it is agreed that timed tests and patient-reported outcome measures should be assessed once per year alongside the Pompe PEDI test in pediatric patients. Muscle strength tests such as Manual Muscle Testing (MMT), the Medical Research Council Scale (MRC), handheld dynamometry, the Fatigue Severity Scale, and age-appropriate assessments for infants and children should be conducted regularly and as needed. For the pediatric population, the following tests have been proposed: MFM20, MFM32, the Walton & Gardner-Medwin Scale, GSGC (Gait, Stairs, Gowers, Chair), Pedi-Pompe, QMFT (Quick Motor Function Test), and AIMS (Alberta Infant Motor Scale). As these tools have only been evaluated by a limited number of experts, we do not consider them to represent formal consensus recommendations. Needle electromyography may be done and repeated as needed.

### Respiratory function

Consensus was reached that pulmonary function assessment using FVC in sitting and supine positions should be strongly recommended, while assessment of maximal respiratory pressures (MIP/MEP) and sleep studies were recommended.

Spirometry is a necessary test and forced vital capacity in sitting and supine positions should be performed 1–2 times per year. In pediatric patients, pulmonary function testing is necessary and—depending on cooperation—the forced vital capacity (FVC%) test should be used. It should be considered to assess minimal and maximal respiratory pressures (MIP/MEP) 1–2 times per year. Sleep studies should be considered and include pulse oximetry, polysomnography (airflow measurement), transcutaneous capnometry and morning arterial blood-gas analysis and it should be considered to secure an age-appropriate setting. It is agreed that nocturnal ventilation leads to improvements in daily life and quality of life for certain Pompe disease patients.

### Cardiology

Consensus was reached that cardiac assessment, including ECG, 24-hour ECG, echocardiography, and chest X-ray, was recommended, while cardiac MRI was considered optional.

Cardiac MRI may be done and repeated as needed. Chest X-ray should be considered and repeated as needed. ECG should be considered and repeated yearly and as needed.

24 h ECG should be considered and repeated as needed. Cardiac echo should be considered and repeated as needed.

### Radiology

Consensus was reached that morphologic muscle imaging in general is strongly recommended, whereas lung/diaphragm MRI and muscle MRI were considered optional. Skeletal X-ray was also considered optional.

Morphologic changes in muscle should be investigated by imaging on a clinically indicated basis. A clinical indication may be baseline assessment in asymptomatic genetic carriers, elevated CK or clinical deterioration in known patients. Glycogen deposition and muscle quality may be assessed by MRI, e.g. in diaphragm and/or other muscles and be repeated yearly and/or as clinically indicated. Also, in patients with high burden, e.g. requiring sedation or experiencing claustrophobia, diaphragm MRI may be indicated. Skeletal X-ray may be done and repeated as needed.

### Gastrointestinal function and diet

Consensus was reached that assessment of body weight and nutritional status, provision of nutrition recommendations or referral to a nutritionist/dietitian, implementation of a specialized diet, use of dietary supplements, liver sonography, and videofluoroscopic swallowing studies are recommended.

Body weight should be assessed regularly and as needed. Nutritionist and/or dietitian referral may be incorporated into monitoring on a clinically indicated basis. For pediatric patients, individualized decision is recommended over a general referral, Further is agreed, that based on current evidence, nutritional supplements may be recommended in patients with specific needs, but not routinely. In patients with osteoporosis, supplement recommendations may be based on bone densitometry results. Videofluoroscopic swallowing assessment should be considered and repeated as needed. Liver ultrasound should be considered and repeated as needed.

### Bone density

Consensus was reached that bone density measurement should be considered (recommendation).

### Cognitive function

By consensus, cognitive and psychological assessment was considered optional and may be repeated as needed.

### Auditory function

Consensus was reached that hearing tests, including otoacoustic emission tympanometry are recommended. They should be repeated as needed.

### Language, speech, and oromotor function

By consensus, Language, speech, and oromotor function may be done and repeated as needed.

### Pain assessment

Consensus was reached that pain history is recommended as part of the standard assessment and systematic pain assessment (e.g. BPI) should be done and repeated if needed.

### Antibody status

By consensus, immunological monitoring was recommended. Immunological parameters (e.g., anti-drug antibodies, IgE) should be considered in all patients, and done upon infusion or allergic reactions upon clinical worsening (e.g., infusion reaction, decline in 6MWT or FVC).

### Rehabilitation and physical therapy and exercise

Consensus was reached that physiotherapy, muscle and respiratory muscle training are recommended generally and rehabilitation programs in patients with progression. It was somewhat agreed that specialized respiratory/muscle training is indicated by literature and patients with neuromuscular diseases should participate in such programs and align these with their follow-up appointments.

### Biochemical markers

Creatine kinase measurement (ck) was recommended by strong consensus and Hex4 was considered optional with a consensus. Ck should be considered for monitoring regularly and/or as clinically indicated. Urinary Hex4 test may be used for monitoring regularly and/or as clinically indicated.

### Relatives’ psychosocial health

By majority agreement assessment of relatives’ psychosocial health was recommended to be included in patient-/caregiver-reported outcome assessments.

### Quality of life (QoL)

By consensus quality of life assessed was recommended. It should be done regularly and as needed, e.g. by EQ-5D-5 L and/or R-Pact.

Recommendations of our group are summarized in Table 1.

## Discussion

Through this national Delphi process, we convened a consensus panel of five Pompe-disease specialists from diverse medical disciplines and five Austrian treatment centers, supplemented by 18 additional clinicians, to establish clear follow-up assessments as the Austrian standard. In two classical online rounds and a subsequent modified third round, across the domains of neuromuscular evaluation, pneumology, biochemical markers, immunology, radiology, diet and nutrition, quality of life, physical therapy/rehabilitation, bone health, and caregiver wellbeing were rated and 34 consensus statements were achieved. These recommendations draw on the existing literature, the practical experiences of participating experts and the structural realities of the Austrian healthcare system. By doing so, they address a critical knowledge gap—particularly salient given the availability of three causal therapies—and provide a foundation for personalized, evidence-informed management of Pompe disease. Beyond its national relevance, our approach and methodological framework have three notable strengths: (i) to our knowledge, this is the only recent consensus to offer AGREE II–compliant [9], clearly weighted recommendations for follow-up assessments in Pompe disease; (ii) it encompasses virtually all domains addressed in the parallel MetabERN [3] clinical pathway project and adds few special aspects; and (iii) it was completed with remarkable efficiency, generating results in just nine months. Given these successes, our methodology offers a promising model for similar consensus processes both within our group and for other expert panels.

Clinical decision-making regarding the initiation, switching, and discontinuation of enzyme replacement therapy (ERT) represents one of the most critical and demanding aspects of managing patients with Pompe disease. To support this, the European Pompe Consortium (EPOC) has defined the “Triple-S” (Start, Switch, Stop) criteria and recommends six-monthly structured clinical monitoring. The recommended minimal set of outcome measures covers the assessment of skeletal muscle and pulmonary function parameters, patient-reported outcomes (PROs) on participation, fatigue, and pain, evaluation of co-morbidities and prognosis, as well as an overall estimate of treatment effectiveness. [4]. The specific parameters were further detailed by a second European expert project, which largely overlapped in authors with the EPOC. Notably, the recommended frequency of many monitoring measures is lower than biannually. In fact, beyond the core assessments—medical history, clinical examination, routine laboratory tests, and anti-drug antibody testing—MMT/MRC, 6-minute walk test (6MWT), timed function tests, PROMs, nutritional status, and pulse oximetry were the only assessments recommended at six-month intervals. Other standard evaluations were suggested at significantly longer intervals or only as needed [3].

This already illustrates that the recommended intervals for individual assessments remain a matter of debate and are highly context dependent. A nationally coordinated approach to these standards offers the opportunity to adapt recommendations to the specific healthcare setting, and our results differ in several aspects from the previously published recommendations. For instance, in our project, none of the assessments were generally recommended more frequently than once per year, which corresponds to the national practice and was generally considered adequate, while acknowledging that deviations from guidelines may be justified in exceptional cases. Although robust evidence to definitively determine the optimal monitoring strategy is lacking, reaching a national consensus on what should be considered standard practice is both reasonable and necessary. This need arises, on the one hand, from the availability of multiple treatment options. The selection of the most suitable therapy is highly dependent on adequate clinical monitoring—whether initiating ERT or switching between therapies. On the other hand, it is crucial that both the decision to start and to stop treatment is based on sound clinical monitoring data [3, 4].

It is therefore evident that inconsistencies in monitoring practices between national centers carry a significant risk of patient confusion, may encourage center-hopping, and further complicate negotiations with healthcare payers in an already challenging therapeutic landscape. Thus, national recommendations on such critical aspects of patient care, as clinical monitoring, are of utmost importance.

However, the development of evidence-based guidelines, which represent the gold standard, is often resource-intensive, time-consuming, and may span several years. As a result, such guidelines may no longer reflect the most current state of knowledge by the time they are published [10]. Expert consensus projects, on the other hand, can be completed more rapidly, but they are susceptible to significant bias if not conducted using a rigorously structured methodology. In particular, the influence of dominant panel members may skew the discussion and outcomes if adequate safeguards are not in place [11].

We therefore chose the structured, formalized process of an S2k guideline [7] to ensure methodological transparency and traceability. This represents a structured expert consensus without systematic literature review. Although support from an industry partner precludes formal recognition as a guideline, a certain degree of representativeness was achieved by involving the clinical centers responsible for Pompe patient care, as well as relevant medical disciplines and professional groups. An S3 guideline was not pursued due to the substantial resource requirements and the lack of comparative studies on the validity of different follow-up assessments.

The clinical assessment procedures discussed are used both for diagnostic purposes and for evaluating the course of disease. In our consensus, we focused on the latter. Generally, such assessments are performed regularly and/or when clinically indicated, for example in the event of clinical deterioration. Regular assessments are typically conducted annually; for more complex or burdensome procedures, longer intervals may be appropriate. The burden of investigations must be carefully weighed against their clinical relevance. Those conducting the investigations should be fully aware of the current relevance and evidentiary status of the respective diagnostic method, and this should be clearly communicated to the patient to allow for shared decision making [12]. The primary aims are the individualized selection of the most appropriate therapy, continuous optimization of treatment and supportive care, confirmation of therapeutic efficacy, and early detection of newly emerging or unexpectedly progressive symptoms to enable timely intervention [3–5, 13]. Clinical assessment and of neuromuscular function (6MWT, timed tests, MMT/MRC, handheld dynamometry, FSS, age-appropriate pediatric tools), respiration and sleep (FVC sitting/supine, MIP/MEP, polysomnography with pulse oximetry and capnometry [14–18]), quantification of biochemical markers (CK, urinary Hex4/Glc4 [19–21]), immunology (anti-drug antibodies/IgE in reaction/worsening) [22–24], imaging for muscle quality assessment and glycogen storage quantification (MRI [25–27], sonography [28]), nutritional evaluation [29–31], quality of life assessment (EQ-5D-5 L) [13, 32–34] and the utilization of patient-reported outcomes (PRO) [15, 35] are generally considered standard [5, 36–38]. There are slight differences between our consensus and the recent review of an expert project of Metab ERN: Apart from the lower frequency of certain examinations described above, additional aspects were also considered. The inclusion of psychosocial care for family members was considered essential, as recent studies clearly demonstrate its central importance for the quality of life and psychological well-being of the entire family [33, 39]. The consideration of physiotherapy and rehabilitation was deemed necessary, as recent evidence has shown that long-term physical activity is associated with improved functional outcomes and should therefore be recommended [40]. Rehabilitation must be regularly adapted and monitored and should therefore be an integral component of routine follow-up assessments [41–47] and psycho-social support for care givers. Bone density assessment by DEXA was considered “nice to have” by Parenti and considered standard for patients with relevant muscle disease by our group.

While there was a clear consensus in our group that age-appropriate test like MFM (Motor Function Measure) 20 Test, MFM32 Test, Walton & Gardner-Medwin Scale, GSGC (Gait, Stairs, Gowers, Chair), Pompe PEDI (Pompe-Paediatric Evaluation of Disability Inventory), QMFT (Quick Motor Function Test) and AIMS (Alberta Infant Motor Scale) [37] should be used, obviously a larger group of specialized pediatricians would have been needed for defining a recommended standard set of tests. Our findings should be interpreted in the context of Austria’s relative low prevalence of diagnosed Pompe patients and the distribution of care across multiple centers [1], which may limit direct generalizability to countries with different settings. However, similar conditions have also been described for much larger countries like France [48] and Spain [49], and optimal coordination of clinical management is of particular importance under such circumstances.

We recognize potential limitations related to participant selection, the relatively low number of experts involved, and the modification of round 3 to an even smaller group. However, efforts were made to include a panel representing all disciplines and centers involved. The restriction of the third round to the Steering Committee was decided in order to obtain comprehensive input on all aspects (with the exception of pediatric aspects) from all participants and thus to efficiently reach concrete decisions. Although this inevitably introduces a certain degree of bias, we consider this approach justified given the full transparency of the process and the fact that all centers and the main involved disciplines were also represented in the third round. Formal validation of consensus stability could have been helpful to systematically substantiate this; however, it was neither planned within the given framework nor feasible retrospectively. While efforts were made to include patient representatives, active patient involvement was not feasible. Industrial support implies that, despite the fact that industry representatives had no influence on the content, the Delphi process does not fully comply with S2k guideline standards in this respect. Although a standardized updating process has not yet been defined, the overall perception of the process was sufficiently positive to suggest that adapting the APOC framework to other rare diseases or expanding it into an S2k ‘living guideline’ appears promising.

## Conclusion

Although our project cannot formally replace a guideline initiated by professional societies and conducted independently of industry, the specific, systematically and structurally derived recommendations hold substantial value for the practical care of patients with Pompe disease. Moreover, our approach qualifies as a highly efficient methodology that may serve as an excellent model for addressing similar clinical questions.

## Data Availability

With the consented statements and the list of provided literature all relevant data and material should be included within the supplementary material. Yet, if justified requests are made we will be open to give further information.
